# Differential Response of Pentanal and Hexanal Exhalation to Supplemental Oxygen and Mechanical Ventilation in Rats

**DOI:** 10.3390/molecules26092752

**Published:** 2021-05-07

**Authors:** Lukas M. Müller-Wirtz, Daniel Kiefer, Joschua Knauf, Maximilian A. Floss, Jonas Doneit, Beate Wolf, Felix Maurer, Daniel I. Sessler, Thomas Volk, Sascha Kreuer, Tobias Fink

**Affiliations:** 1 CBR—Center of Breath Research, Department of Anaesthesiology, Intensive Care and Pain Therapy, Saarland University Medical Center, Homburg, 66421 Saarland, Germany; daniel.kiefer@uks.eu (D.K.); joschua.knauf@gmx.de (J.K.); max.floss@outlook.de (M.A.F.); jonas.doneit@gmx.de (J.D.); beate.wolf177@yahoo.de (B.W.); felix.maurer@uks.eu (F.M.); thomas.volk@uks.eu (T.V.); sascha.kreuer@uks.eu (S.K.); tobias.fink@uks.eu (T.F.); 2 Outcomes Research Consortium, Cleveland, OH 44195, USA; ds@or.org; 3 Department of Outcomes Research, Anesthesiology Institute, Cleveland Clinic, Cleveland, OH 44195, USA

**Keywords:** mechanical ventilation, anesthesia, supplemental oxygen, oxygen toxicity, lipid peroxidation, volatile aldehydes, pentanal, hexanal, volatile organic compounds

## Abstract

High inspired oxygen during mechanical ventilation may influence the exhalation of the previously proposed breath biomarkers pentanal and hexanal, and additionally induce systemic inflammation. We therefore investigated the effect of various concentrations of inspired oxygen on pentanal and hexanal exhalation and serum interleukin concentrations in 30 Sprague Dawley rats mechanically ventilated with 30, 60, or 93% inspired oxygen for 12 h. Pentanal exhalation did not differ as a function of inspired oxygen but increased by an average of 0.4 (95%CI: 0.3; 0.5) ppb per hour, with concentrations doubling from 3.8 (IQR: 2.8; 5.1) ppb at baseline to 7.3 (IQR: 5.0; 10.8) ppb after 12 h. Hexanal exhalation was slightly higher at 93% of inspired oxygen with an average difference of 0.09 (95%CI: 0.002; 0.172) ppb compared to 30%. Serum IL-6 did not differ by inspired oxygen, whereas IL-10 at 60% and 93% of inspired oxygen was greater than with 30%. Both interleukins increased over 12 h of mechanical ventilation at all oxygen concentrations. Mechanical ventilation at high inspired oxygen promotes pulmonary lipid peroxidation and systemic inflammation. However, the response of pentanal and hexanal exhalation varies, with pentanal increasing by mechanical ventilation, whereas hexanal increases by high inspired oxygen concentrations.

## 1. Introduction

High inspired oxygen concentrations may cause toxicities, including oxidative stress, hyperoxic vasoconstriction, and resorption atelectasis [1,2]. Furthermore, reactive oxygen species promoted by high oxygen concentrations attack cell components, including lipids, proteins, and DNA—all of which provoke local and systemic inflammation [3]. While prolonged hyperoxia undoubtedly causes lung damage, the extent of hyperoxia-induced injury during short-term mechanical ventilation, such as might occur during surgery, is controversial [2].

The lowest inspired oxygen concentration used for intraoperative mechanical ventilation is about 30%, usually resulting in only slight hyperoxemia because mechanical ventilation causes a degree of shunt and dead-space ventilation. Nevertheless, higher inspired oxygen concentrations are frequently used, either out of necessity to maintain a suitable arterial oxygen saturation, or simply to provide pulmonary oxygen reserve in the case of an airway problem. To assess the clinical tradeoff between additional safety and potential hyperoxia-induced lung injury, the effects of different inspired oxygen concentrations on pulmonary oxidative stress and systemic inflammation are thus of considerable interest.

Lipid peroxidation is a major mechanism by which oxygen causes toxicity [4]. The process releases volatile products, including the two aldehydes, pentanal and hexanal [5], both of which have been proposed as possible breath biomarkers for pulmonary pathologies, such as lung cancer [6,7] and ventilator-induced lung injury [8]. Hyperoxia produces reactive oxygen species in lung tissue with a consequent increase in lipid peroxidation [9,10]. Other volatile lipid peroxidation products, especially ethane and pentane, consistently increase in the breath of hyperoxic animals and humans [11,12,13,14]. However, the influence of inspired oxygen on pentanal and hexanal exhalation in mechanically ventilated subjects remains to be determined for a valid interpretation of these newly proposed biomarkers.

We, therefore, evaluated pentanal and hexanal exhalation in rats mechanically ventilated for 12 h with various inspired oxygen concentrations. We simultaneously determined interleukin serum concentrations as a measure of systemic inflammation. Specifically, we tested the primary hypothesis that high- and medium-inspired oxygen concentrations provoke more pentanal and hexanal exhalation than lower concentrations in rats. Secondarily, we tested the hypothesis that high inspired oxygen concentrations increase serum cytokine concentrations.

## 2. Results

### 2.1. Experimental Conditions

**Figure 1:**
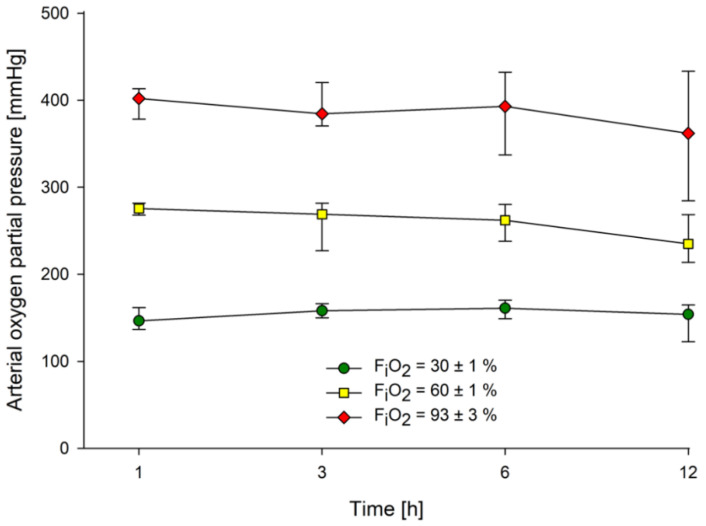
Arterial oxygen partial pressure. Data presented as medians and interquartile ranges. F_i_O_2_ = fraction of inspired oxygen.

All animals survived the study period and were included. The median weight of the rats was 343 (IQR: 336; 351) g, and all survived the 12 h observation period. Heart rate and mean arterial pressure decreased over the observation period, but similarly at each inspired oxygen concentration (Supplementary File 1; Figure S1). Blood gas values, hemoglobin, electrolytes, glucose, and lactate remained within physiological ranges (Supplementary File 1, Table S1). Median minute ventilation was 180 (IQR: 174; 184) ml/min, median peak pressure was 10.9 (IQR: 10.6; 11.1) cmH_2_O, and median tidal volume was 2.8 (IQR: 2.7; 2.8) ml over all groups. Median arterial partial pressures of oxygen differed markedly among the groups, as expected (Figure 1).

### 2.2. Breath Analysis

**Figure 2:**
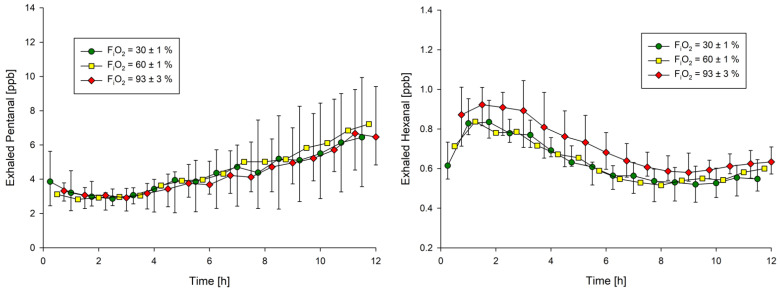
Pentanal and hexanal exhalation over 12 h of mechanical ventilation. Data presented as medians and interquartile ranges. F_i_O_2_ = fraction of inspired oxygen. Exhaled concentrations were measured at 15-minute intervals, with every third value displayed and error bars omitted for 60% to enhance clarity.

Exhaled pentanal did not differ as a function of inspired oxygen but increased over all groups by an average of 0.4 (95%CI: 0.3; 0.5) ppb per hour of mechanical ventilation. The median exhaled pentanal concentration therefore almost doubled from 3.8 (IQR: 2.8; 5.1) ppb at baseline to 7.3 (IQR: 5.0; 10.8) ppb after 12 h of mechanical ventilation (Table 1, Figure 2).

Exhaled hexanal was slightly higher in rats exposed to 93% inspired oxygen, with average concentrations being 0.09 (95%CI: 0.002; 0.172) ppb higher than in rats ventilated with 30% inspired oxygen. Exhaled hexanal initially increased in all groups, reached a maximum after around 2 h, and stabilized at a lower plateau after approximately 6 to 12 h; concentrations did not increase over time (Table 1, Figure 2).

### 2.3. Systemic Inflammation

**Figure 3:**
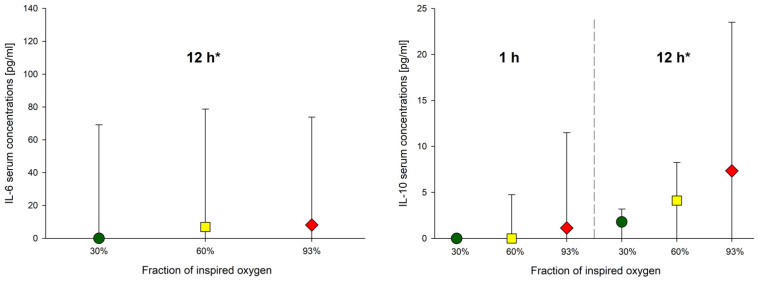
Cytokine serum concentrations. Data presented as medians and interquartile ranges. IL-6 at 1 h was 0 for all animals. IL-6 concentrations did not significantly differ by inspired oxygen (*p* = 0.888), whereas IL-10 concentrations were significantly greater at 60% and 93% compared to 30% inspired oxygen (30% vs. 60%: *p* = 0.035; 30 vs. 93%: *p* = 0.001). Interleukin serum concentrations across all groups increased significantly between 1 and 12 h of mechanical ventilation (* IL-6: *p* = 0.002, * IL-10: *p* = 0.035).

IL-6 concentrations did not significantly differ as a function of inspired oxygen fraction (*p* = 0.888), whereas IL-10 concentrations averaged 2.5 (95%CI: 0.2; 4.8) pg/mL higher at 60% and 7.2 (95%CI: 2.8; 11.5) pg/mL higher at 93% than with 30% inspired oxygen (*p* = 0.035, *p* = 0.001; Figure 3). Interleukin serum concentrations across all groups increased significantly between 1 and 12 h of mechanical ventilation (IL-6: *p* = 0.002, IL-10: *p* = 0.035, Figure 3).

## 3. Discussion

We expected the exhalation of both pentanal and hexanal to increase at high inspired oxygen concentrations. Instead, responses differed, with hexanal exhalation increasing by high inspired oxygen concentrations, whereas pentanal exhalation gradually increased over 12 h of mechanical ventilation, but unrelated to inspired oxygen.

Hyperoxia produces reactive oxygen species in lung tissue with a consequent increase in lipid peroxidation [9,10]. Hexanal exhalation was consistently greater at high inspired oxygen concentrations. Likewise, previous studies reported increased exhalation of ethane and pentane in hyperoxic individuals [11,12,13,14]. Thus, available data suggest that high-inspired oxygen concentrations induce pulmonary lipid peroxidation. Breath analysis may, therefore, facilitate the early detection of hyperoxic lung injury, and may be especially helpful for investigating specific treatments that potentially reduce hyperoxic lung injury.

Mechanical ventilation increased pentanal exhalation over time, with concentrations almost doubling over just 12 h. As lung distension promotes injury [15,16], we used tidal volumes of 8 mL/kg, which falls within the broadly accepted range of 6–9 mL/kg commonly used in humans [17] and rodents [18,19]. However, even with moderate tidal volumes, mechanical ventilation damages the cell membrane and activates cellular repair mechanisms [20], which include transferring lipids to the cell membrane to enlarge the cell surface and help maintain its integrity [21]. Sufficiently high tidal volumes can overcome cellular repair mechanisms, leading to membrane defects, followed by apoptosis or necrosis [20]. Cell death seems unlikely with the ventilator settings we used, but sublethal cellular membrane damage may expose polyunsaturated fatty acids to oxidative processes, and thus increase pentanal exhalation. This theory is consistent with in vitro studies, showing that pentanal is the predominant volatile aldehyde generated by oxidizing lipid membranes exposed to mechanical stress through sonication [22] and by oxidizing isolated polyunsaturated fatty acids [23].

The observed gradual increase in pentanal exhalation over 12 h of mechanical ventilation is consistent with our previous finding that exhaled pentanal is highly sensitive to volutrauma [8]. Based on the biological background presented above, we previously postulated that stretch-induced cell membrane damage exposes membrane lipids to oxidative processes, thereby increasing the exhalation of pentanal. In contrast, hexanal concentrations did not increase over 12 h, although hexanal and pentanal are likely generated by similar mechanisms [5]. The relatively low vapor pressure of hexanal may have contributed [24,25], but it is non-obvious why hexanal did not increase. Future studies may clarify reasons for the differential response of pentanal and hexanal to mechanical ventilation.

Inspired oxygen concentrations greater than 30% did not increase exhaled pentanal, probably because even that amount of oxygen is sufficient to oxidize all available membrane lipids from which pentanal arises. Our lowest inspired oxygen concentration was 30% since lower concentrations are rarely used for mechanical ventilation. Our results, therefore, well characterize the effects of supplemental oxygen during mechanical ventilation. The current study consistently showed that pentanal increases due to mechanical ventilation and adds the important finding that exhaled pentanal is a potential breath biomarker that can be interpreted independently from inspired oxygen.

Pentanal and hexanal are potential biomarkers of lung, breast, and gastrointestinal cancers in humans [6,7,26,27]. Cancer causes cell death, for example, by tumor growth, exceeding supply with nutrients, attacks by immune cells, or by the inflammation and destruction of surrounding tissues. Cell death is accompanied by cell membrane breakdown, exposing lipids to oxidation, and may thus prompt the exhalation of aldehydes. As might, therefore, be expected, lung cancer increases pentanal and hexanal exhalation [6,7]. Furthermore, acute respiratory distress syndrome increases both pentanal and hexanal concentrations in blood [28]. Taken together, pentanal and hexanal seem to be general markers of cell membrane damage, and our findings suggest that exhaled pentanal also increases when lung tissue is mechanically stressed.

Interleukin serum concentrations increased over 12 h of mechanical ventilation, with only IL-10 being greater at higher inspired oxygen concentrations. Helmerhorst et al. reported that IL-6 serum concentrations remain similar at various inspired oxygen concentrations over 12 h of mechanical ventilation in mice, but IL-10 concentrations in bronchoalveolar fluid substantially increase [16]. However, others reported no influence of oxygen on the mRNA expression of IL-10 in mouse lungs [29,30]. Consistent with inflammation accruing over time, lung tissue IL-6 mRNA increases after 48 h of hyperoxia [31]. High inspired oxygen concentrations induce cytokine gene expression in alveolar macrophages of surgical patients, reflecting local pulmonary inflammation [32]. However, IL-6 was less affected by hyperoxia than other cytokines, and IL-10 was not measured [32]. Taken together, the available data suggest that high-inspired oxygen induces a slight systemic inflammatory response during up to 12 h of mechanical ventilation.

The most obvious limitation of our study is that lipid peroxidation, cell membrane components, and antioxidative capacities presumably differ among species. However, lipid peroxidation is fundamental to oxidative stress. It is, therefore, likely that results in humans are qualitatively similar, although presumably quantitatively different. We did not conduct a formal sample-size estimate because the treatment effect was non-obvious. We also note that anesthetic drugs could have influenced our results, as propofol has antioxidative properties and may inhibit lipid peroxidation processes [33,34,35]. We did not include a control group ventilated with 21% oxygen because, in previous studies, this concentration resulted in hypoxemia and even death. Finally, our results are specific to mechanical ventilation; results likely differ with spontaneous ventilation.

In summary, mechanical ventilation and high inspired oxygen promote pulmonary lipid peroxidation and systemic inflammation. However, the response of pentanal and hexanal exhalation varies, with pentanal increasing in response to mechanical ventilation and hexanal increasing in response to high concentrations of inspired oxygen. Our results suggest that exhaled pentanal, a potential biomarker for lung injury, can be interpreted independent of the inspired oxygen concentration during mechanical ventilation.

## 4. Materials and Methods

### 4.1. Animals

Experiments were conducted in accordance with the German Animal Welfare Act and with approval from the responsible Institutional Animal Care and Use Committee (Landesamt für Soziales, Saarland, Saarbrücken, Germany, No. 28/2018, date of approval: 01.08.2018).

Thirty male Sprague Dawley rats (280–380 g body weight, age 8–10 weeks) were obtained from Charles River Laboratory International (Sulzfeld, Germany) and kept in our institutional animal facility under controlled conditions (temperature 20 ± 2 °C and 50 ± 5% relative humidity). The rats had free access to water and standard pellet food. Monitoring and preparation were performed, as previously described [36]. No specific inclusion or exclusion criteria were applied. Animals were included as long as the experimental protocol was adequately followed and in the absence of poor welfare signs (e.g., wounds, secretion of harderian gland, signs of dehydration, diarrhea, isolation from others, itching).

### 4.2. Anesthesia

Anesthesia was induced with sevoflurane (Baxter, Unterschleißheim, Germany) and maintained with intravenous propofol (Fresenius Kabi, Bad Homburg, Germany) starting at 25 mg/kg/h with hourly reductions of 0.5 mg/h until a minimal rate of 15 mg/kg/h was reached. Ketamine (Rotexmedica, Trittau, Germany) was added with 25 mg/kg/h throughout the experiment for analgesia. Neuromuscular blockade was induced by a bolus of 10 mg/kg rocuronium (Grünenthal, Stolberg, Germany) and maintained by a continuous infusion of 25 mg/kg/h rocuronium. Animals were observed for 12 h and then killed by exsanguination.

### 4.3. Ventilation

Oxygen was produced by a concentrator (Compact 525, Devilbiss, NY, USA) with a maximum output of 93 ± 3%, mixed with generated nitrogen (Genius, Peak Scientific, Inchinnan, Scotland, UK) and purified by activated charcoal filtration. Inspired oxygen was constantly monitored and adapted to maintain concentrations at 30% or 60% within a range of ± 1% (sensor: GGA 370, device: GMH 3695, Greisinger, Regenstauf, Germany). For the highest oxygen group, the maximum output of the oxygen concentrator was used, resulting in a concentration of 93 ± 3%. Ten rats each were randomly assigned to three different fractions of inspired oxygen: 30%, 60%, and 93%.

Animals were randomized 1:1:1 based on a computer-generated list. Investigators were not blinded during the experiments, but allocation was concealed during post-experimental analysis. Animals were ventilated with a tidal volume of 8 mL/kg, a respiratory rate of 63 breaths/min, and a PEEP of 2 cmH_2_O (VentStar small animal ventilator, RWD Life Sciences, Shenzhen, China). The respiratory rate was reduced by 10% when the partial pressure of carbon dioxide was less than 28 mmHg. Similarly, the respiratory rate was increased by 10% when partial pressure exceeded 45 mmHg.

### 4.4. Breath and Blood Samples

Blood for gas analyses was sampled after 1, 3, 6, and 12 h to monitor ventilation (Radiometer ABL 800 Basic, Willich, Germany). Ten milliliters of exhaled air were sampled and analyzed with two multi capillary columns—ion mobility spectrometers (MCC-IMS by B&S Analytik, Dortmund, Germany) in 15-minute intervals, as previously described [36]. The MCC-IMS was calibrated by pentanal and hexanal standards ranging from 0.1 to 50 ppb (analytical standard, Merck, Darmstadt, Germany), as previously described [23]. Arterial blood samples of 600 µL were collected after 1 and 12 h. Plasma was stored at −75 °C. Interleukin 6 and 10 serum concentrations were measured by enzyme-linked immunosorbent assay (ELISA). Positive controls of each cytokine were measured routinely with each assay (ELISA Antibodies BD OptEIA; BD Biosciences Pharmingen, San Diego, CA, USA).

### 4.5. Statistics

Statistical analyses were carried out with R 4.0.2 (R Core Team, 2020) using the packages *geepack* (Højsgaard, Halekoh, and Yan, 2006) and *broom* (*v0.7.5*; Robinson, Hayes and Couch, 2021). Figures were created with SigmaPlot 12.5 (Systat Software GmbH, Erkrath, Germany). Normality was assessed by visual inspection of histograms and quantile-quantile plots. Most data were not normally distributed. Therefore, all results are presented as medians and interquartile ranges. Influences of inspired oxygen and mechanical ventilation time on aldehyde exhalation were assessed by linear generalized estimating equations regression to account for within-subject correlations. The influence of inspired oxygen on interleukin concentrations was assessed by linear generalized estimating equations regression combined with a Wald statistic. Interleukin concentrations after 1 and 12 h were compared over all groups by a Wilcoxon signed-rank test. A two-sided *p* < 0.05 was considered statistically significant. There was no a priori sample size estimate since the expected effect sizes and the clinical significance of increases in aldehyde exhalation through supplemental inspired oxygen are essentially unknown.

## Figures and Tables

**Table 1:** Influence of inspired oxygen and mechanical ventilation on pentanal and hexanal exhalation.

**Pentanal**			
**Parameter**	**Regression Coefficient**	**95% Confidence Interval**	***p***
F_i_O_2_ = 93%	0.03	−1.4–1.4	0.967
F_i_O_2_ = 60%	0.67	−1.1–2.4	0.454
F_i_O_2_ = 30%	0	-	-
Ventilation time [h]	0.4	0.3–0.5	<0.001
**Hexanal**			
**Parameter**	**Regression Coefficient**	**95% Confidence Interval**	***p***
F_i_O_2_ = 93%	0.09	0.002–0.172	0.046
F_i_O_2_ = 60%	0.03	−0.06–0.116	0.506
F_i_O_2_ = 30%	0	-	-
Ventilation time [h]	−0.01	−0.016–(−0.007)	<0.001

Linear generalized estimating equations (GEE)—regression was performed. The regression coefficient of ventilation time refers to one hour of mechanical ventilation. F_i_O_2_ = fraction of inspired oxygen.

## Data Availability

Data are contained within the article and Supplementary Material. The data on pentanal and hexanal exhalation are available in Supplementary File 2 and on interleukin serum concentrations in Supplementary File 3.

## Supplementary Materials

The following are available online, Supplementary File 1 (including Figure S1: Vital parameters and Table S1: Results of blood gas analysis); Supplementary File 2: Pentanal and hexanal exhalation; Supplementary File 3: Interleukin serum concentrations.

## Sample Availability

Samples of the compounds are not available from the authors.
