# Supplementary material for: Differential Response of Pentanal and Hexanal Exhalation to Supplemental Oxygen and Mechanical Ventilation in Rats
Source: Molecules. 2021 May 7;26(9):2752. doi: 10.3390/molecules26092752 (PMC8124567; doi:10.3390/molecules26092752)
Supplement: Supplementary file 1 [file molecules-26-02752-s001.zip › molecules-1209346-supplementary/Supplementary File 1.pdf]

Supplementary File 1:

Differential response of pentanal and hexanal exhalation to supplemental oxygen and mechanical ventilation in rats

Lukas M. Müller-Wirtz [1,3], Daniel Kiefer [1], Joschua Knauf [1], Max Floss [1], Jonas Doneit [1], Felix Maurer [1,3], Beate Wolf [1], Daniel I. Sessler [2,3], Thomas Volk [1,3], Sascha Kreuer [1,3], Tobias Fink [1,3]

1) **C**enter of **B**reath **R**esearch, Department of Anesthesiology, Intensive Care and Pain Therapy, Saarland University Medical Center, Homburg (Saar), Germany

2) Department of **O**UTCOMES **R**ESEARCH, Anesthesiology Institute, Cleveland Clinic, Cleveland, OH, USA

3) **O**UTCOMES **R**ESEARCH Consortium, Cleveland, Ohio, USA.

Corresponding Author:

Dr. med. Lukas Martin Müller-Wirtz

CBR - Center of Breath Research

Department of Anesthesiology, Intensive Care and Pain Therapy

Saarland University Medical Center and Saarland University Faculty of Medicine

66421 Homburg / Saar, Germany

Phone: +4968411622785

Email: lukas.wirtz@uks.eu

ORCID: 0000-0002-7984-1798

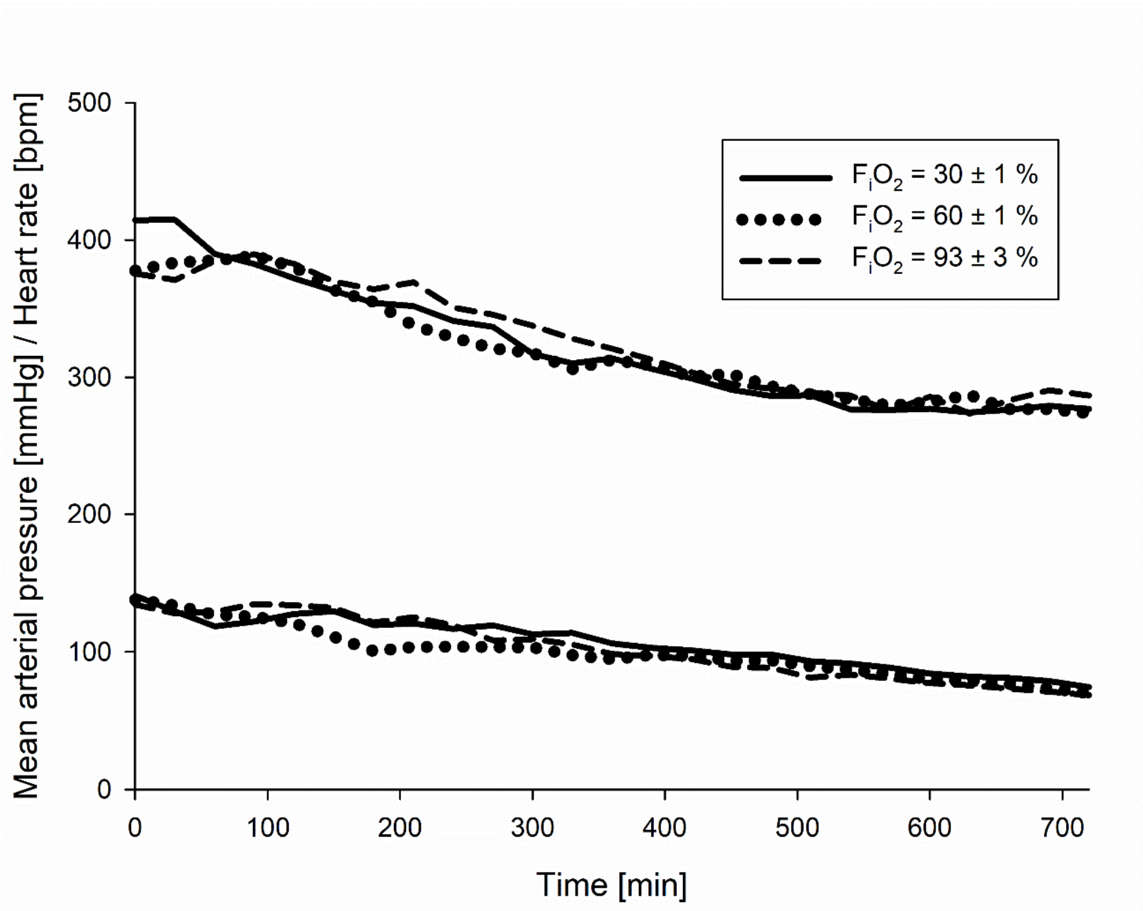

Figure S1: Vital parameters

Upper curves represent heart rate and lower curves represent mean arterial pressure. Data presented as medians per groups.

Table S1: Results of blood gas analysis

	Group 1 (Inspiratory oxygen fraction: 30%)				Group 2 (Inspiratory oxygen fraction: 60%)				Group 3 (Inspiratory oxygen fraction: 93%)			
**Time**	**1 h**	**3 h**	**6 h**	**12 h**	**1 h**	**3 h**	**6 h**	**12 h**	**1 h**	**3 h**	**6 h**	**12 h**
**pH**	**7.46**<br>7.42-7.47	**7.45**<br>7.43-7.48	**7.44**<br>7.4-7.46	**7.4**<br>7.37-7.43	**7.42**<br>7.4-7.44	**7.42**<br>7.42-7.44	**7.44**<br>7.41-7.45	**7.39**<br>7.36-7.41	**7.43**<br>7.41-7.45	**7.43**<br>7.39-7.47	**7.42**<br>7.38-7.43	**7.37**<br>7.29-7.42
**pCO_2**<br>[mmHg]	**41**<br>39-44	**35**<br>34-37	**35**<br>32-37	**36**<br>33-38	**44**<br>41-45	**39**<br>36-41	**39**<br>34-40	**40**<br>37-41	**45**<br>42-46	**39**<br>38-40	**37**<br>36-41	**39**<br>38-42
**pO_2**<br>[mmHg]	**147**<br>137-162	**158**<br>150-166	**161**<br>149-170	**154**<br>123-165	**276**<br>268-282	**269**<br>227-282	**262**<br>238-280	**235**<br>214-269	**402**<br>378-413	**385**<br>371-420	**393**<br>337-432	**362**<br>285-433
**Hb**<br>[g/dL]	**13.2**<br>12.6-13.8	**11.6**<br>10.7-11.9	**10.8**<br>10.1-10.9	**10.2**<br>9.2-10.6	**12.9**<br>12.1-13.8	**11.8**<br>11.5-12	**10.8**<br>10.3-11.4	**9.8**<br>9.6-10.6	**12.8**<br>12.4-13.5	**11.9**<br>11.2-12.4	**10.9**<br>10.5-11.7	**9.5**<br>9.1-10.8
**sO_2**<br>[%]	**97**<br>97-98	**98**<br>97-98	**98**<br>97-98	**98**<br>97-98	**98**<br>98-100	**99**<br>98-100	**99**<br>98-100	**99**<br>99-100	**99**<br>99-99	**99**<br>99-99	**99**<br>99-99	**99**<br>98-99
**K^+**<br>[mmol/L]	**4.5**<br>4.4-4.6	**3.9**<br>3.7-4.2	**3.9**<br>3.7-4.2	**4.6**<br>4.3-4.7	**4.8**<br>4.3-4.8	**4.2**<br>3.9-4.4	**4.2**<br>3.9-4.4	**4.2**<br>3.8-4.6	**4.5**<br>4.2-4.8	**4.1**<br>3.7-4.6	**4.3**<br>3.8-4.6	**4.5**<br>4-5.2
**Na^+**<br>[mmol/L]	**141**<br>141-141	**145**<br>143-146	**144**<br>143-147	**142**<br>141-143	**142**<br>140-143	**147**<br>144-147	**147**<br>145-150	**146**<br>142-147	**141**<br>141-143	**144**<br>142-144	**143**<br>141-146	**142**<br>139-145
**Ca^{2+}**<br>[mmol/L]	**1.1**<br>1-1.2	**1.1**<br>1.1-1.2	**1.1**<br>1.1-1.2	**1.2**<br>1.1-1.2	**1.2**<br>1.1-1.3	**1.2**<br>1.2-1.2	**1.2**<br>1.1-1.2	**1.2**<br>1.1-1.2	**1.3**<br>1.2-1.3	**1.2**<br>1.1-1.3	**1.1**<br>1.1-1.2	**1.1**<br>1.1-1.2
**Glucose**<br>[mg/dL]	**136**<br>126-145	**125**<br>121-133	**120**<br>112-124	**101**<br>(96-108)	**130**<br>123-140	**127**<br>116-139	**124**<br>104-129	**102**<br>98-108	**141**<br>131-143	**132**<br>124-147	**123**<br>115-128	**97**<br>95-114
**Lactate**<br>[mmol/L]	**0.3**<br>0.2-0.5	**0.7**<br>0.6-1	**0.7**<br>0.5-0.9	**0.5**<br>0.2-0.5	**0.5**<br>0.4-0.6	**0.9**<br>0.5-1.3	**0.9**<br>0.6-1.3	**0.6**<br>0.5-0.6	**0.3**<br>0.1-0.4	**0.7**<br>0.5-0.8	**0.7**<br>0.5-0.9	**0.4**<br>0.3-0.5
**BE**<br>[mmol/L]	**4.4**<br>3.5-6.2	**1.5**<br>-0.6-2.3	**-1.2**<br>-3-0.1	**-2.6**<br>-4.3-(-1.1)	**3.8**<br>3-4.6	**1.1**<br>-0.4-2	**0.4**<br>-0.5-1.8	**-1.3**<br>-2.8-(-0.7)	**5.6**<br>3.2-6.6	**1.5**<br>0.1-3.3	**-0.6**<br>-2.1-1.2	**-2.7**<br>-5.6-0.5
**HCO_3**<br>[mmol/L]	**28**<br>27-29	**26**<br>24-27	**24**<br>23-25	**23**<br>21-24	**27**<br>27-28	**25**<br>24-26	**25**<br>24-26	**24**<br>22-24	**29**<br>27-30	**26**<br>25-27	**24**<br>23-26	**22**<br>20-25

Data presented as medians and interquartile ranges.
